# Adaptive evolution of *oriC* through *in vitro* propagation of a mini-chromosome in RCR

**DOI:** 10.1093/nar/gkaf772

**Published:** 2025-08-11

**Authors:** Shota Suzuki, Masayuki Su’etsugu

**Affiliations:** Department of Life Science, College of Science, Rikkyo University, 3-34-1 Nishi-Ikebukuro, Toshima-ku, Tokyo 171-8501, Japan; Moderna Enzymatics Co., Ltd, 2-3-8 Shinkiba, Koto-ku, Tokyo 136-0082, Japan; Department of Life Science, College of Science, Rikkyo University, 3-34-1 Nishi-Ikebukuro, Toshima-ku, Tokyo 171-8501, Japan

## Abstract

Diversification and selective propagation are the main driving forces of evolution, resulting in the emergence of organisms possessing various strategies. Here, we conducted *in vitro* evolution of the replication origin (*oriC*) under the pressure of an AT-rich mini-chromosome amplification using a reconstituted *Escherichia coli* replication-cycle reaction (RCR). Using next-generation sequencing, we identified that these evolved *oriC*s contain select mutations within the duplex unwinding element (DUE) and DnaA-binding site (DnaA box) regions, which are crucial for replication initiation. Real-time detection of RCR amplification (real-time RCR) revealed that the DUE mutations, which decreased the GC content, along with the introduction of a specific A/T sequence in DUE-M and a consecutive KAK (K = T or G) motif in DUE-R, enhanced the RCR amplification efficiency compared to the wild-type *oriC* (*oriC*wt). A competitive amplification assay also elucidated that the DnaA box mutations confer a competitive advantage over coexistent *oriC*wt. Although these DUE and DnaA box mutations were selected through the same amplification reaction, they exhibited distinct competitive amplification strategies. The DUE mutant represents a faster propagation strategy (*r*-strategy), while the DnaA box mutant represents an adaptive strategy with a competitive advantage (*K*-strategy), representing the *r*/*K*-selection theory in molecular evolution.

## Graphical Abstract

**Graphical Abstract:**
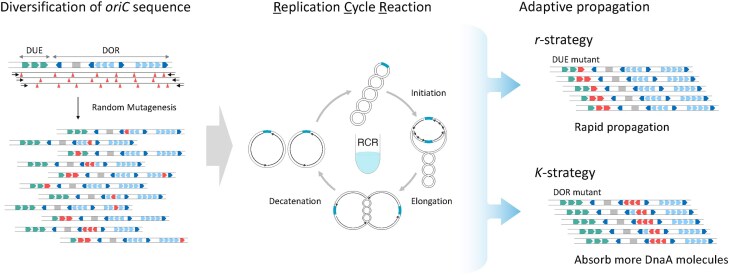

## Introduction

Molecular evolution is driven by diversification and selective propagation. Mutations in DNA, the fundamental genetic material, are vital for directing diversification and generating genetic variability among organisms. The DNA sequences that encode more competitive and adaptive genetic information than others are favored through natural competition and selection, resulting in their predominance. This evolutionary strategy can be applied to improve proteins [1–4]. Studies have used random mutagenesis to generate DNA diversity and select adapted genes and the products by mimicking this natural process. Using these molecular evolutionary methods, many protein variants can be screened for desired functions with a simple experiment. Additionally, *in vitro*, replication systems present a unique opportunity to explore potential molecular evolutionary pathways leading to the emergence of novel adapted forms [5–8]. These experiments have demonstrated the ability to replicate ecological evolution, such as Darwinian evolution, *in vitro*.

**Figure 1.**
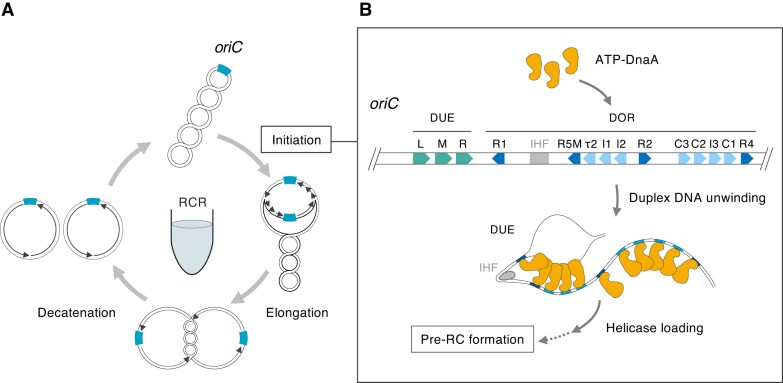
Replication-cycle reaction (RCR). (**A**) Schematic representation of the RCR amplification cycle. The process begins at the replication origin (*oriC*) and propagates circular DNA exponentially through bidirectional amplification under isothermal conditions, producing supercoiled DNA. The initiation step only proceeds when the duplex DNA is unwound at *oriC*. (**B**) Schematic representation of the initiation process at *oriC*. The structure of *oriC* consists of an AT-rich duplex unwinding element (DUE) and an initiator protein DnaA-assembly region (DOR). ATP–DnaA binds to the DOR and forms a homo-multimeric complex. This complex, in cooperation with IHF, induces unwinding of the DUE. The unwound region allows loading of the DnaB helicase, which leads to the formation of the pre-replication complex. Green arrows show the DUE-L, -M, and -R regions. Blue arrows show DnaA boxes: light blue indicates DnaA–ATP binding sites and deep blue indicates both DnaA–ATP and DnaA–ADP binding sites. The gray box indicates an IHF binding site.

DNA replication, crucial for transferring genetic information to future generations, consists of initiation, elongation, and termination. DNA replication in most bacteria such as *Escherichia coli* (Fig. 1A) is initiated at the chromosomal replication origin (*oriC*). The DNA polymerase synthesizes new DNA strands bidirectionally using two existing DNA strands as the template at a rate of ∼750 bases per second [9]. Replication terminates at a region opposite to *oriC*. Replicated DNAs are separated and form negatively supercoiled monomers competent for initiation at *oriC*. The initiation step of the DNA replication cycle is rate limiting because it is tightly regulated to prevent over-initiation [10, 11].

The entire replication cycle of the *E. coli* chromosome has been reconstituted *in vitro* using purified components [12]. Subsequently, we previously achieved efficient continuous and autonomous DNA replication using minimal 25 individual proteins *in vitro*, known as RCR [13]. The RCR replicates bidirectionally and exponentially propagates a supercoiled mini-chromosome (*oriC*-carrying plasmids) through autonomous cycle repetition in an isothermal reaction. Using RCR, high-fidelity amplification with an error rate of 1.2 × 10^−8^ errors per base per cycle was achieved [13]. The RCR enables the amplification of a mini-chromosome from circularly assembled linear fragments [13] and the propagation of various mini-chromosomes, including GC-rich DNA (9.3 kb, 69% GC) [14], large F-plasmids (230 kb) [14], and 1–2 Mb-long *E. coli* chromosomes [15]. Therefore, it is a useful tool for amplifying various supercoiled DNAs without *in vivo* cloning.

Replication initiation mechanisms from *oriC* have been well studied in *E. coli* [16–18]. A 245-bp minimal *oriC* region contains a DUE flanked by a DnaA-oligomerization region (DOR) and a DNA bending protein IHF binding site (Fig. 1B) [18–21]. The binding of the initiator protein DnaA to its binding site (DnaA box) in the DOR and the IHF to its binding site triggers replication initiation, leading to the unwinding of the AT-rich DUE, enabling the entry of the DnaB helicase (Fig. 1B) [20, 22–27]. DnaA forms a stable complex with ATP or ADP, resulting in an active ATP–DnaA complex and an inactive ADP–DnaA complex [28, 29]. Eleven DnaA boxes exist within the minimal *oriC* region, three of which (R1, R2, and R4) have the consensus sequence 5′-TGTGnA^T^/_A_AA-3′ and high affinity for DnaA [30–32]. The remaining eight sites have one to six variations resulting in low affinity for DnaA, and only the active ATP–DnaA can bind to these DnaA boxes [31, 33, 34]. These bound ATP–DnaAs form homo-multimeric complexes [25, 35–37], and the tension generated during complex formation is believed to lead to the duplex opening at DUE, allowing replisome assembly in this region [38, 39]. Modifying low-affinity DnaA boxes, such as τ2–I1–I2 and C3–C2–I3, to high-affinity ones has been shown to promote the binding of DnaA including ADP-DnaA to *oriC* and unwinding of DUE, resulting in over-replication *in vivo* [40], which suggested that the modification of DnaA box enhances DNA replication initiation. Whereas the DUE consists of three tandem repeats of a 13-mer sequence with the consensus sequence 5′-GATCTnTTnTTTT-3′ [20], and defined ATP–DnaA ssDNA (single-stranded DNA) binding sites, AGATCT [41] and TT[A/G]T(T) [27, 35]. *In vitro* DnaA binding experiments have shown that the thymine-rich leading (upper) strand of the right DUE (DUE-R) exhibits strong interaction with ATP–DnaA [24, 27, 35, 42], and mutational analyses have demonstrated that DUE-R is essential for duplex DNA unwinding [42, 43]. *Bacillus subtilis oriC* and other bacterial *oriC* have repeating trinucleotide motifs called DnaA-trios (3′-NAN-5′; consensus 3′-GAT-5′) adjacent to a DnaA box that are essential for unwinding DUE and binding DnaA to ssDNA [44, 45]. *Escherichia coli oriC* also contains three DnaA-trio motifs (5′-TAT TAG GAT-3′) adjacent to DUE-R.

In this study, while amplifying AT-rich mini-chromosomal DNA, we serendipitously discovered that mutations in the *oriC* DUE region significantly increased the RCR replication rate. Building upon this observation, we employed an evolutionary strategy to generate more effective *oriC* mutants. Resultantly, we obtained evolved *oriC*s with additional mutations in the DnaA box and DUE regions. Analysis of these mutations revealed that low-affinity DnaA boxes (τ2, I1, and I2) became more similar to the DnaA consensus sequence. Meanwhile, the DUE region exhibited decreased GC content, accompanied by the formation of a specific A/T sequence in DUE-M and a consecutive KAK (K = T or G) motif in DUE-R. Although mutations in both the DnaA box and DUE regions provide a competitive advantage in RCR amplification, each region exhibits a distinct strategy, resembling the *r*/*K*-selection theory in ecology [46, 47]. The DUE mutations represent a rapid propagation strategy (*r*-strategy), while the DnaA box mutations represent an adaptive strategy with a competitive advantage (*K*-strategy), suggesting the applicability of the *r*/*K*-selection theory in molecular evolution.

## Materials and methods

### RCR

RCR replication reactions were conducted as previously described with slight modifications [13, 48]. A 5-μl RCR reaction mixture containing 1× RCR buffers I and II, 1× replication enzyme (RE) mix, and 3 ng/μl λ phage DNA was incubated at 33°C for 12 h. The RCR reactions were diluted using 1× RCR buffer and incubated at 30°C for 30 min. An aliquot was mixed with Stop buffer [48] and analyzed via agarose gel electrophoresis (AGE). To enhance the amplification of the AT-rich mini-chromosome, we used an AT-RCR in which 300 nM HU and 100 nM H-NS were added to the RCR mixture to stabilize the amplification of an AT-rich mini-chromosome. HU and H-NS are DNA-binding proteins [49–51], and H-NS exhibits preferential binding affinity for AT-rich sequences [52, 53]. For the low-temperature amplification assay, a 10-μl RCR reaction mixture comprising 1× RCR buffers I and II, 0.5× RE mix, and 0.05% Tween 20 was incubated at 33°C for 12 h. Multiple DNA assembly reactions were conducted using a commercial Cell-Free Cloning System (OriCiro Genomics, Tokyo, Japan) based on the manufacturer’s instructions. The assembled products were diluted 1:10 for the RCR reaction.

### Agarose gel electrophoresis

AGE was performed as previously reported [14]. In this study, 1% gels were made using high-quality agarose (Recenttec) and run for 1 h at a constant voltage of 65 V in 0.5× Tris–borate–EDTA (TBE) buffer. The gels were stained with dsGreen (Funakoshi) and scanned using a Typhoon FLA 9500 (GE Healthcare) or Fusion Solo S (Vilber).

### Construction of *oriC* mutants

The details regarding the construction of *oriC* mutants on the mini-chromosomal DNAs and the oligo DNAs used in this study are provided in Supplementary data and  Supplementary Table S1. PCR amplification was performed using KOD One polymerase (TOYOBO, Osaka, Japan). The resulting fragments were gel extracted and purified using column purification (Macherey-Nagel, Düren, Germany). The resulting *oriC* mutant fragments were assembled into pSV-β-galactosidase (pSVβ; Promega, Madison, WI, USA) vector (7.2 kb) using Cell-Free Switching System (OriCiro Genomics, Tokyo, Japan) according to the manufacturer’s instructions. The resultant amplicons were purified using column purification (Macherey-Nagel, Düren, Germany). *oriC*dueM9A, *oriC*dueM9T, *oriC*dueR13A, *oriC*dueR13T, *oriC*dueR4AT, and *oriC*dueRKAK were constructed using a conventional *E. coli* cloning strategy as described in Supplementary data.

### Screening of evolved *oriC* mutants

According to the manufacturer’s instructions, we prepared a random *oriC* library using error-prone PCR with the GeneMorph II Random Mutagenesis Kit (Agilent). Briefly, the *oriC* region was amplified using the primer P55/P56. The resulting fragments were purified by gel extraction using a DNA column purification kit (NucleoSpin Gel and PCR Clean-up, Macherey-Nagel, Düren, Germany). Purified *oriC* fragments were assembled with a 10-kb Region II fragment (23% GC) using a Cell-Free Cloning System (OriCiro Genomics, Tokyo, Japan) and amplified in AT-RCR.

### Next-generation sequencing analysis

The next-generation sequencing (NGS) analyses of the amplified circular DNAs were performed using iSeq 100 (Illumina) and NEBNext Ultra II FS DNA Library Prep with Sample Purification Beads and Multiplex Oligos for Illumina (NEB). The analysis of single nucleotide polymorphism (SNP) was identified using Geneious Prime (Biomatters Ltd).

### Real-time RCR

Real-time RCR was performed as previously described with slight modifications [54]. Each reaction was performed using a 10-μl reaction mixture containing a final concentration of 1× RCR buffers I and II, 0.05% Tween 20, 300 nM YO-PRO-1 iodide (em. 491/ ex. 509) (Thermo Fisher Scientific), and 0.5× RE mix. The 9-μl reaction mixture was pre-incubated at 20°C for 15 min and then replaced on ice to prevent initiation of the RCR reaction. Subsequently, 1 μl of *oriC* DNA was added at a final concentration of 1 pM, and the RCR reaction was initiated at 30°C using the Real-Time System (Thermal Cycler Dice Real Time System, Takara, Japan). The amplified DNA was detected every 10 min using an SYBR filter. The doubling time of the amplified DNA in the RCR system was calculated from a slope of the logarithmic phase of the amplification curve. To ensure standardized experimental conditions for each *oriC* mutant, all mutants were cloned into a pSVβ plasmid with a GC content of 52%.

### P1 nuclease assay

The P1 nuclease assays were performed following the methods described in previous studies [27]. Briefly, the *oriC*5.6AT plasmid or *oriC*wt (0.63 nM) was mixed with 32 nM IHF and varying amounts of the ATP-bound form of DnaA. The mixtures were incubated at 38°C for 3 min in 10 μl of buffer P, which consisted of 90 mM HEPES–KOH (pH 7.6), 0.1 mM zinc acetate, 8 mM magnesium acetate, 30% glycerol (v/v), and 0.32 mg/ml bovine serum albumin, and supplemented with 5 mM ATP and 125 mM potassium chloride. The reactions were then treated with 2 μl of P1 nuclease (0.7 units, Wako) for an additional 3 min. For the reactions at 30°C and 34°C, the preincubation was also performed at the respective temperatures. To terminate the reactions, 4 μl of Stop buffer containing 1% sodium dodecyl sulphate and 25 mM ethylenediaminetetraacetic acid was added. The resulting DNA was then subjected to 1% AGE, stained with dsGreen (Funakoshi), and visualized using a Fusion Solo S system (Vilber). Quantification of the unwound plasmid products was done using ImageJ software [55].

### Competitive amplification assay

A 13-kb mini-chromosome containing *oriC*wt was used as a competitor and mixed with a 7-kb mini-chromosome containing *oriC* mutant at a ratio of 200:100 pM. The DNA mixture was amplified using RCR with slight modifications. A 10-μl reaction mixture was used containing 1× final concentration of RCR buffers I and II, 0.05% Tween 20, and 0.5× RE mix. The RCR reaction mixture was pre-incubated at 20°C for 15 min and then mixed with the DNA mixture on ice to prevent RCR initiation. After adding the DNA mixture, the RCR was performed at 30°C for 12 h. The RCR mixture was diluted by 1× RCR buffer and incubated at 30°C for 30 min mixed with Stop buffer [48]. An aliquot was mixed with Stop buffer and analyzed by AGE (65 V, 90 min).

## Results

### *oriC* DUE mutations enable RCR amplification of AT-rich mini-chromosome

**Figure 2.**
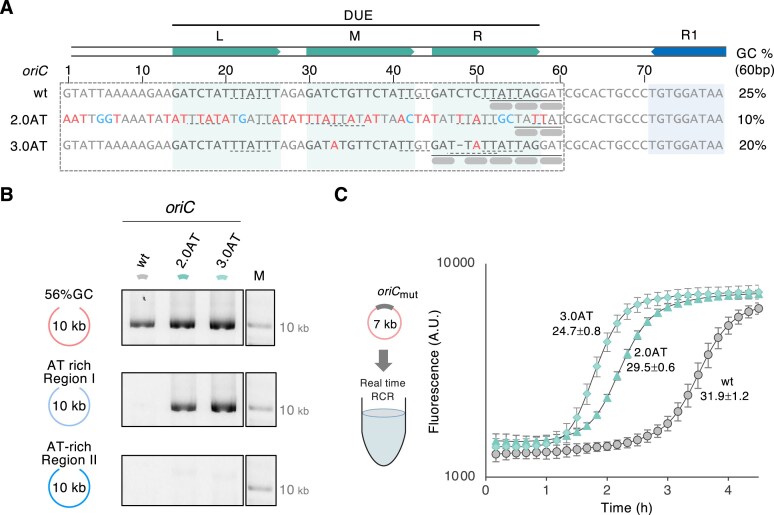
*oriC* DUE mutations enhancing RCR amplification. (**A**) DUE sequences of *oriC*2.0AT (2.0AT), *oriC*3.0AT (3.0AT), and *oriC*wt (wt). Pale green and pale blue boxes indicate the DUE-L, -M, and -R regions and the DnaA box R1, respectively. The underlines represent continuous KAK sequences in the DUE of wt and 3.0AT or the DUE-like region of 2.0AT, and the thick gray bars indicate the KAK motif. The dotted underlines show the DnaA ssDNA binding sequence TT[A/G]T(T) [27, 35]. Gray characters denote bases identical to the native sequence, and red (A/T) or blue (G/C) characters denote substitutions. The hyphen indicates a base deletion. The gray dotted box indicates the region used for calculating the GC content, as shown on the right. The respective base numbers are counted from 70 bp upstream of the first base of the DnaA box R1. (**B**) Partial improvement in AT-rich DNA amplification using *oriC*2.0AT and *oriC*3.0AT. Three 10-kb fragments [a 56% GC region, Region I (21%GC), and Region II (23%GC)] were assembled with *oriC*2.0AT (2.0AT), *oriC*3.0AT (3.0AT), and *oriC*wt (wt) and amplified using RCR. The resulting RCR products were then analyzed by 1% AGE in 0.5x TBE and stained with dsGreen. Supercoiled DNA size marker (M) was run on the same gel. (**C**) Real-time RCR of *oriC*2.0AT and *oriC*3.0AT. The amplification of 7-kb circular DNA containing each oriC was performed in the presence of the YO-PRO-I intercalator, initiated from a DNA concentration of 1 pM at 30°C. The names of the *oriC* mutants are labeled on the individual amplification curves, and the doubling times with the standard deviations are shown below. The experiment was performed three times, and the error bars indicate the standard error of the mean (A.U., arbitrary units).

In the course of experiments to synthesize mini-chromosomal DNAs using RCR and *E. coli oriC*, we attempted to construct a minimal *Mycoplasma* genome, JCVI-syn3A (syn3) DNA, with a GC content of 24%. After several attempts to construct AT-rich mini-chromosomes with a part of syn3, we coincidentally obtained amplified products of ∼40 kb from two regions: the 2AT region (total size = 50 kb; GC content = 24%) and the 3AT region (total size = 40 kb; GC content = 26%) (Supplementary Fig. S1A and B). To confirm the sequence, we performed NGS analysis using these amplified products, revealing that the *oriC* regions in 2AT and 3AT products contain DUE mutations (Fig. 2A). The mutations observed in 2AT, a part of the syn3 DNA sequence, resulted from the deletion of a 7-kb region extending from the juxta DUE region of *oriC* to the inside of the JCVISYN3_0693 gene region, which is not a replication-related gene in syn3. This deletion occurred within the first fragment of 2AT (Supplementary Fig. S1B). Several TTAT(T) sequences were observed in the DUE-like region of the 2AT product that resemble the previously reported DnaA ssDNA binding sequence motif, TT[A/G]T(T) [27, 35], suggesting that this sequence could potentially function as DUE (Fig. 2A). Whereas the 3AT product had two C to A substitutions and a single base deletion in the DUE, likely caused by PCR error, increasing the number of KAK (K = T or G) motifs from three to five in the DUE-R (Fig. 2A). These mutations resulted in a low GC content in the DUE region (GC contents of DUE between base numbers 1 and 60: wild type, 25%; 2AT, 10%; and 3AT, 20%) (Fig. 2A).

To investigate the effect of the *oriC* mutations on the amplification of AT-rich mini-chromosomes, we constructed two *oriC* mutants containing DUE-like and DUE mutations found in the 2AT and 3AT products, named *oriC*2.0AT and *oriC*3.0AT, respectively (Fig. 2A). Subsequently, we performed RCR amplification using the assembled products of the *oriC* mutant fragments (0.4 kb in length) and 10-kb Region I (21% GC) and Region II (23% GC) fragments from the syn3 genome, using the Cell-Free Cloning System (OriCiro). The Region I fragment was the lowest GC content 10 kb region of syn3 (Supplementary Fig. S1A), while the Region II fragment corresponds to the first region of 2AT, in which a 7-kb deletion was observed during the amplification of the 2AT region, as described above. The results of RCR amplification showed that *oriC*2.0AT and *oriC*3.0AT enabled amplification of the Region I fragment (Fig. 2B). Contrastingly, neither of the *oriC* mutants amplified the Region II fragment. Although the DUE mutations did not completely improve the amplification efficiency of these regions, these results indicated that the DUE mutations have the potential to facilitate the amplification of an AT-rich mini-chromosome.

### *oriC*2.0AT and *oriC*3.0AT enhance the amplification rate

We hypothesized that a DUE region with lower GC content might facilitate the unwinding of the duplex DNA, enhancing replication initiation. To test the hypothesis, we evaluated the amplification efficiency of *oriC*2.0AT and *oriC*3.0AT using real-time detection of RCR amplification (real-time RCR) where the amplified DNA was detected using the intercalator YO-PRO-1. In this experiment, we constructed mini-chromosomes containing *oriC* mutants using the pSVβ 7-kb circular DNA (52% GC) to assess the amplification efficiency of *oriC*, including the wild-type *oriC* (*oriC*wt). The amplification rates were determined by the doubling time, which was calculated from the slope of the logarithmic plot of the amplification. The real-time RCR analysis showed an increased amplification rate of *oriC*2.0AT (29.5 min) and *oriC*3.0AT (24.7 min) by 1.1-fold and 1.3-fold, respectively, compared to that of *oriC*wt (31.9 min) (Fig. 2C). Additionally, the amplification curve rose earlier for *oriC*2.0AT and *oriC*3.0AT, suggesting improved amplification efficiency during the initial phase of RCR amplification. These results indicate that the lower GC content of the DUE region enhanced the amplification rate. We previously reported a doubling time of ∼8 min for RCR amplification using a 9.5-kb mini-chromosome containing *oriC*wt [13], which indicates that YO-PRO-1 inhibits RCR amplification, as previously reported [54]. Moreover, despite the lower GC content in its DUE, the amplification rate of *oriC*2.0AT (10% GC) was slower than that of *oriC*3.0AT (20% GC). These findings suggest that sequence specificity and decreased GC content in the DUE region contribute to enhance the amplification rate.

### Evolutionary engineering of *oriC* under the selective pressure of AT-rich mini-chromosome amplification

**Figure 3.**
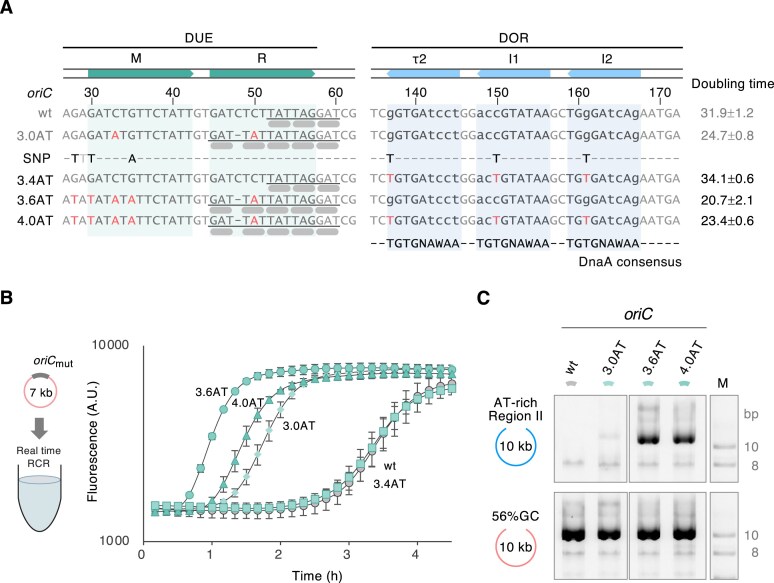
Isolation of evolved *oriC* through evolutionary molecular engineering. (**A**) DUE and DOR sequences of *oriC*3.4AT, *oriC*3.6AT, and *oriC*4.0AT with mutations selected through evolutionary molecular engineering. The selected mutations from ep-*oriC*3.0AT are shown in the SNP row, where gray and black characters represent bases partially and perfectly identical to the SNP, respectively. The hyphens in the SNP row indicate that no nucleotide was selected. Gray or red characters denote bases identical or not identical to the native sequence, respectively. In the sequences of 3.0AT, 3.6AT, and 4.0AT, hyphens indicate base deletions. Lowercase characters denote bases that are not identical to the DnaA consensus sequence (indicated in the bottom line of the DOR region). Pale green boxes represent the 13-mers in the DUE-M and DUE-R regions, while pale blue boxes indicate the DnaA box sequences τ2, I1, and I2. The respective base numbers are counted from 70 bp upstream of the first base of the DnaA box R1. The underline represents the continuous KAK sequence in DUE-R, and the thick gray bars indicate the KAK motif. The doubling time of amplification (min), shown on the right with the standard deviation, was calculated from panel (B). (**B**) Real-time RCR of *oriC*3.4AT, *oriC*3.6AT, and *oriC*4.0AT. A 7-kb circular DNA containing each *oriC* was amplified in the presence of the YO-PRO-I intercalator, initiated from a DNA concentration of 1 pM at 30°C. The names of the *oriC*s are indicated next to the individual amplification curves. The experiment was performed three times, and the error bars indicate the standard error of the mean (A.U., arbitrary units). (**C**) Improvement in AT-rich DNA amplification using *oriC*3.6AT and *oriC*4.0AT. Two 10-kb fragments [a 56% GC region and Region II (23%GC)] were assembled with *oriC*3.0AT (3.0AT), *oriC*3.6AT (3.6AT), *oriC*4.0AT (4.0AT), and *oriC*wt (wt) and amplified using RCR. The resulting RCR products were then examined by 1% AGE in 0.5x TBE and stained with dsGreen. Supercoiled DNA size marker (M) was run on the same gel.

To obtain an improved *oriC* that enhances the amplification rate, we employed evolutionary molecular engineering using RCR. We performed random mutagenesis on the entire *oriC* region using error-prone DNA polymerase-based PCR, resulting in mutagenized *oriC* libraries: *oriC*wt (ep-*oriC*wt), *oriC*2.0AT (ep-*oriC*2.0AT), and *oriC*3.0AT (ep-*oriC*3.0AT). As previously mentioned, we obtained improved *oriC*s by amplifying a previously nonamplifiable AT-rich DNA. Therefore, we assembled these mutagenized *oriC* libraries with the 10-kb Region II fragment of syn3 DNA, which could not be amplified before (Fig. 2B). We amplified them using AT-RCR in triplicate reactions. Notably, no amplified products were observed under normal RCR conditions. We obtained three products from ep-*oriC*2.0AT and two from ep-*oriC*3.0AT, but none from ep-*oriC*wt. We performed NGS and SNP analysis on the amplified products to identify the mutations responsible for improved amplification. The analysis revealed several mutations within the DUE-M, -R, and the left of DnaA boxes τ2, I1, and I2 (Supplementary Fig. S2). The DUE mutations were both transition and transversion mutations from GC to AT and lowering the GC content of DUE, which suggested facilitating the duplex DNA unwinding. Meanwhile, identical DnaA box mutations were found at τ2–I1–I2 positions in many or all amplified products (Supplementary Fig. S2). These three DnaA box mutations were G137T in τ2, C150T in I1, and G161T in I2. These mutations render the τ2–I1–I2 sequences more similar to the DnaA consensus sequence (5′-TGTGnA^T^/_A_AA-3′) (Fig. 3A) [30–32]. DnaA domain IV recognizes the left six bases of the major groove and the right three bases of the minor groove [56]. Therefore, the DnaA box mutations might potentially facilitate the interaction between initiator DnaA and τ2–I1–I2 DnaA boxes.

### Evolved *oriC* DUE mutations further improve RCR amplification

We next constructed an evolved *oriC*, named *oriC*4.0AT, by introducing select mutations within the DUE and DnaA box regions into the fast-amplifying *oriC*3.0AT (Fig. 3A). Real-time RCR analysis revealed that the amplification rate of *oriC*4.0AT (23.4 min) was 1.4-fold and 1.1-fold faster than those of *oriC*wt (31.9 min) and *oriC*3.0AT (24.7 min), respectively (Fig. 3A and B), confirming that the mutations enhanced the RCR amplification. To investigate whether *oriC*4.0AT enhanced the amplification rate due to mutations in DnaA boxes or DUE, we constructed *oriC*3.4AT and *oriC*3.6AT, containing mutations either in the τ2–I1–I2 region or in the DUE, respectively (Fig. 3A). *oriC*3.6AT (20.7 min) exhibited a higher amplification rate (1.1-fold) and an earlier rising point of the amplification curve compared to *oriC*4.0AT (Fig. 3A and B). In contrast, the amplification rate of *oriC*3.4AT (34.1 min) decreased to 0.7-fold compared to that of *oriC*4.0AT and its amplification curve showed a similar rising point to that of *oriC*wt (Fig. 3A and B). These results suggest that the DUE mutations significantly enhance the amplification rate, whereas DnaA box mutations in the τ2–I1–I2 region may slightly decrease the amplification rate. Additionally, *oriC*3.6AT demonstrated the ability to amplify AT-rich DNA that was previously nonamplifiable, exhibiting a higher amplification efficiency than *oriC*3.0AT (Fig. 3C). Compared to the DUE of *oriC*3.0AT, the mutated *oriC*3.6AT DUE contains repetitive AT sequences on the left of DUE-M (Fig. 3A) and the continuous KAK sequence in DUE-R, suggesting that these sequences contribute to the enhancement of RCR amplification.

### Validation of specific sequences within the DUE and DnaA boxes that enhance RCR amplification

**Figure 4.**
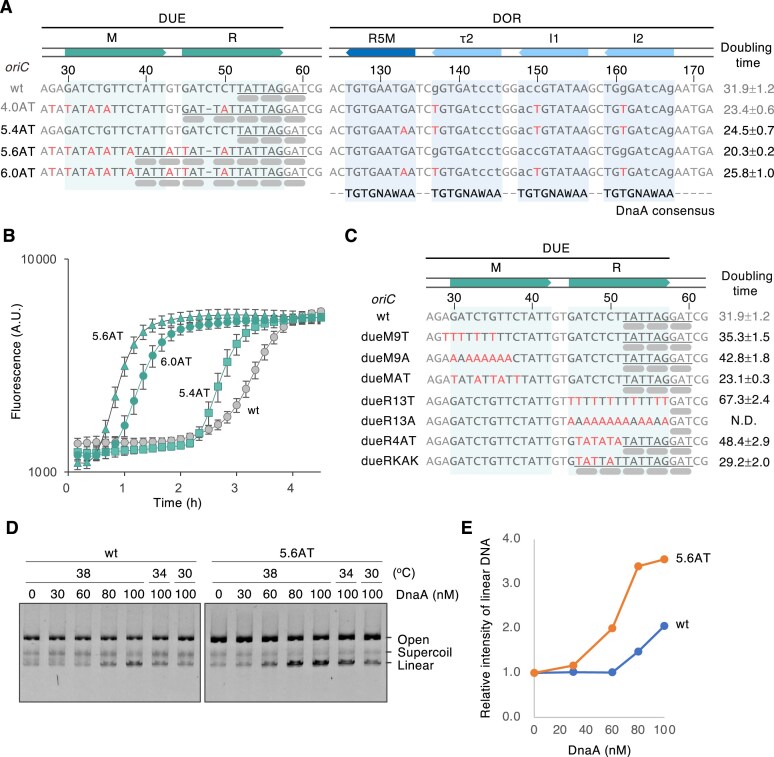
Effect of designed DUE and DnaA box mutations on RCR amplification (**A**) DUE and DOR sequences of *oriC*5.4AT, *oriC*5.6AT, and *oriC*6.0AT possessing designed mutations in the DUE and DnaA boxes. The continuous KAK sequence in the DUE-R is underlined, and the thick gray bars indicate the KAK motif. Pale green boxes represent the DUE-M and DUE-R regions, while pale blue boxes indicate the DnaA box sequences R5M, τ2, I1, and I2. Gray and red characters represent bases that are identical or not identical to the native sequence, respectively. Hyphens in sequences indicate a base deletion, and lowercase characters denote sequences not identical to the DnaA consensus sequence (indicated in the bottom line of the DOR region). The respective base numbers are counted from 70 bp upstream of the first base of the DnaA box R1. The doubling time of amplification (min), shown on the right with the standard deviation, was calculated from panel (B). (**B**) Real-time RCR of *oriC*5.4AT, *oriC*5.6AT, and *oriC*6.0AT. Amplification of 7-kb circular DNA containing each *oriC* was carried out in the presence of the YO-PRO-I intercalator, initiated from a DNA concentration of 1 pM at 30°C. The names of the *oriC*s are indicated on the side of the amplification graph. The experiment was performed three times, and the error bars indicate the standard error of the mean (A.U., arbitrary units). (**C**) DUE sequences of *oriC*dueM9T, *oriC*dueM9A, *oriC*dueMAT, *oriC*dueR13T, *oriC*dueR13A, *oriC*dueR4AT, and *oriC*dueRKAK. Pale green boxes indicate the DUE-M and DUE-R regions. Continuous KAK sequence in the DUE-R is underlined, and the thick gray bars indicate the KAK motifs. Gray and red characters represent bases that are identical or not identical to the native sequence, respectively. The doubling time (min) of amplification is shown on the right. Standard deviations were calculated from three independent experiments. N.D. indicates that DNA amplification was not detected. (**D**) P1 nuclease assay of *oriC*wt and *oriC*5.6AT. Supercoiled *oriC* plasmids (1.3 nM, 7 kb) were incubated with the indicated amounts of ATP–DnaA in the presence of IHF (36 nM), followed by co-incubation with P1 nuclease. The experiment was performed three times (*n* = 3), and a representative result was shown because consistent results were obtained. (**E**). Quantification of unwinding activity of *oriC*wt and *oriC*5.6AT. The relative amount of the P1 nuclease-digested *oriC* DNA per input DNA molecule is shown as the relative intensity of linear DNA, calculated from panel (D).

Based on the previous results, we hypothesized that introducing additional mutations into the DUE region to decrease the GC content and increase the number of KAK sequences may further enhance the amplification rate. Furthermore, we speculated that converting the low-affinity R5M sequence, which is important for initiating DnaA homo-oligomer formation [37], to the sequence identical to the DnaA box consensus may enhance DnaA binding to *oriC*. To test this hypothesis, we added further mutations into *oriC*4.0AT and constructed *oriC*6.0AT, which has DUE mutations for the two additional KAK sequences and a DnaA box mutation for the DnaA box consensus type of R5M mutation (Fig. 4A). The substitution for additional KAK in *oriC*6.0AT makes the DUE GC content decrease to 10%. The amplification rate of *oriC*6.0AT (25.8 min) was slightly slower than that of *oriC*4.0AT (23.4 min) (Fig. 4A and B). When only the DUE mutations of *oriC*6.0AT were assessed (*oriC*5.6AT), the amplification rate became faster than that of *oriC*6.0AT (20.3 min; Fig. 4A). This result supports the previous observation that the higher-affinity version of DnaA box mutations has a negative effect on the RCR amplification rate. In terms of the amplification rate, no significant differences were observed between *oriC*5.6AT and *oriC*3.6AT. *oriC*5.4AT (24.5 min; Fig. 4A), containing only the DnaA box R5M–τ2–I1–I2 mutations of *oriC*6.0AT, exhibited a delay of the rising point of the amplification curve, while its amplification rate was almost similar, compared to that of *oriC*6.0AT.

To determine whether an increase in A/T sequences alone in the DUE-MR region could result in the enhancement of RCR amplification, we examined DUE-M and DUE-R mutants (Fig. 4C). The real-time RCR results revealed that *oriC* mutants with a replacement of DUE-M sequences to poly-A (dueM9A; 42.8 min) or poly-T (dueM9T; 35.3 min) amplified with the doubling time slower than that of *oriC*wt (31.9 min). We then selected an efficient DUE-M sequence in RCR amplification from an *oriC* library in which the DUE-M 13-mer was replaced with 13-mer poly-W (W = A or T). NGS analysis gave that the candidate DUE-M sequence was 5′-TatAtTAtTtatt-3′ (uppercase: substituted sequences). The amplification rate of an *oriC* mutant with this substituted DUE-M sequence (dueMAT; 23.1 min) was faster than that of *oriC*wt, indicating that some sequence specificity of A/T bases plays an important role. The amplification rate of a DUE-R mutant in which the sequence was replaced with poly-T (dueR13T; 67.3 min) was markedly slower than that of *oriC*wt, while a mutant with a poly-A replacement (dueR13A) failed to amplify in the RCR system. Replacement of the DUE-R region with repetitive AT sequences, which are known to confer DNA flexibility [57], resulted in a modest improvement, although the amplification rate remained lower than *oriC*wt (dueR4AT; 48.4 min). Notably, introducing two consecutive KAK motifs—extending the wild-type-derived KAK repeat to a total of five—without significantly altering the base composition from dueR4AT, further accelerated the amplification rate beyond that of *oriC*wt (dueRKAK; 29.2 min). These results demonstrate that a continuous KAK sequence promotes efficient RCR amplification.

We performed a P1 nuclease assay on *oriC*5.6AT to confirm the promoting effect of DUE mutations on specific *oriC* duplex DNA unwinding. We used 7-kb mini-chromosomes containing *oriC*wt and *oriC*5.6AT (Fig. 4D). Because P1 endonuclease cleaves ssDNA, it can nick both strands of the unwinding *oriC*, resulting in the production of linear DNA. The result indicated that *oriC*5.6AT could unwind duplex DNA under lower DnaA concentrations and lower temperatures than that of *oriC*wt (Fig. 4D and E). These results show that DUE mutations in *oriC*5.6AT enhance *oriC* unwinding in a DnaA-dependent manner.

### Designed DUE containing seven repetitions of the KAK motif in DUE-R promotes RCR amplification at low temperature

**Figure 5.**
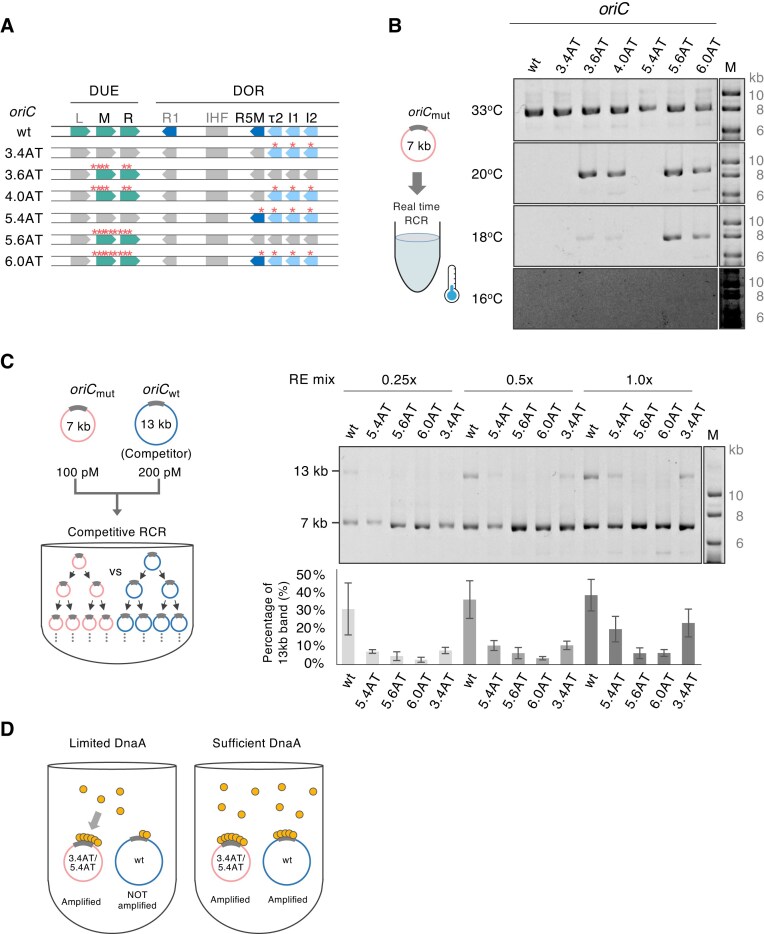
Impact of DUE and DnaA box mutations on RCR amplification at low temperature and in a competitive amplification assay (**A**) Schematic representation of the mutations in *oriC*3.4AT, *oriC*3.6AT, *oriC*4.0AT, *oriC*5.4AT, *oriC*5.6AT, and *oriC*6.0AT, as used in panels (B) and (C). Red asterisks indicate the positions of mutated bases. Colored and gray boxes denote mutated and nonmutated regions, respectively. Green arrows indicate the DUE-L, -M, and -R regions. Blue arrows indicate DnaA boxes: light blue represents DnaA–ATP binding sites, and deep blue represents both DnaA–ATP and DnaA-ADP binding sites. (**B**) Low-temperature RCR amplification. 7-kb DNA containing each *oriC* was amplified for 12 h at the indicated temperature. Amplified products were assessed by 1% AGE. The experiment was performed twice (*n* = 2), and a representative result was shown because consistent results were obtained. Supercoiled DNA size marker (M) was run on the same gel. (**C**) Competitive amplification assay. The amplification of 7-kb circular DNA containing each *oriC* was carried out in the presence of 13-kb circular DNA containing *oriC*wt, used as a competitor. The 7-kb and 13-kb DNAs were mixed at a ratio of 100:200 pM and amplified in an RCR reaction mixture containing the indicated final concentration of RE mix. Amplified products were separated by 1.0% AGE. Supercoiled DNA size marker (M) was run on the same gel. The bottom graph shows the residual percentage (%) of 13-kb DNA, calculated from the band intensity calculated using NIH ImageJ software. The experiment was performed three times, and the error bar indicates the standard error of the mean. (**D**) Schematic representation of the competitiveness model for DnaA box mutants. Since *oriC*3.4AT (3.4AT) and *oriC*5.4AT (5.4AT) bind more strongly to DnaA, they can absorb more free DnaA than *oriC*wt. This effect is more pronounced under limited DnaA conditions, and DnaA box mutants are preferentially amplified over *oriC*wt.

Since the evolved DUE in *oriC*3.6AT (Fig. 3A) and designed DUE in *oriC*5.6AT (Fig. 4A) have low GC content, we expected these DUE regions to enhance duplex DNA unwinding more efficiently than that of *oriC*wt and could improve low-temperature amplification. To test this, we conducted RCR amplification using 1 pM of a 7-kb mini-chromosome containing *oriC* variant for 12 h at lower temperatures (16°C–33°C). The results demonstrated that *oriC*5.6AT and *oriC*6.0AT were successfully amplified at 18°C, while *oriC*3.6AT and *oriC*4.0AT showed only slight amplification at the same temperature (Fig. 5A and B). Additionally, 16°C was the lowest temperature at which amplification of *oriC*5.6AT and *oriC*6.0AT was observed. However, the amplified products of *oriC*3.4AT and *oriC*5.4AT containing only DnaA box mutations were not observed at temperatures below 20°C. These results suggest that DUE mutations are particularly effective in promoting RCR amplification under low-temperature conditions, and that the continuous KAK sequence in the DUE-R region may contribute to this enhancement.

### DnaA box mutations in τ2–I1–I2 impart a competitive advantage, representing a *K*-strategy

Mutations that render the sequence more similar to the DnaA consensus sequence increase the affinity for DnaA. Consequently, in coexistence with *oriC*wt, these mutants may have evolved by competitively sequestering DnaA. To test this hypothesis, we performed a competitive amplification assay using a mixture of 7-kb mini-chromosomal DNA containing *oriC* mutants with 13-kb mini-chromosomal DNA containing *oriC*wt (as a competitor) (Fig. 5C). Because larger mini-chromosomal DNAs are amplified more slowly in RCR [54], the initial mix ratio (100 pM *oriC* mutants and 200 pM *oriC*wt) was used to facilitate band comparison on an agarose gel and to minimize potential RCR size biases. For *oriC*3.4AT (τ2–I1–I2 mutations) and *oriC*5.4AT (R5M–τ2–I1–I2 mutations), >20% of the 13-kb *oriC*wt DNA was observed, whereas in *oriC*5.6AT and *oriC*6.0AT, the proportion of 13 kb of *oriC*wt was ∼6%. When the RE mix was reduced to 0.25×, the 13-kb *oriC*wt maintained a proportion of ∼30%, whereas *oriC*3.4AT and *oriC*5.4AT were decreased to ∼7.5%. These results support a model in which evolved DnaA box mutants competitively sequester DnaA from 13-kb *oriC*wt under conditions in which the availability of DnaA required for replication initiation is limited (Fig. 5D). Taken together, these results demonstrate distinct strategies in the competitive amplification of DUE and DnaA box mutations. The DUE mutations follow the *r*-strategy, whereas the DnaA box mutations follow the *K*-strategy; thus supporting the applicability of the *r*/*K*-selection theory to molecular evolution.

## Discussion

In this study, we performed an evolutionary experiment on *oriC* using RCR and obtained two types of *oriC* mutants: DUE and DnaA box mutants. Each of the mutants exhibited distinct mechanisms to gain an advantage over the *oriC*wt during DNA amplification. The DUE mutations accelerate the amplification rate by facilitating *oriC* unwinding, whereas the DnaA box mutations resulted in a competitive advantage over the *oriC*wt because of their higher affinity for DnaA. This *in vitro* experiment mimics the *r*/*K* selection theory described in ecology.

RCR is a valuable cell-free tool to amplify large circular DNA [13, 15]. Another important finding of this study was an improvement in RCR amplification efficiency by the *oriC* mutation. Indeed, the *oriC*5.6AT mutant showed the following advantages for RCR than *oriC*wt: faster amplification rate, amplification at lower temperature (16°C), and amplification of AT-rich backbone sequence. These features will expand the application of circular DNA amplification in RCR.

The DUE mutations were repeatedly selected from the AT-rich (syn3) DNA amplification experiment, as shown in *oriC*3.0AT, and through random *oriC* library screening. However, the DnaA box mutations were selected only in the random *oriC* screening. Therefore, DUE mutations seem more likely to occur than DnaA box mutations. The DUE mutations increased the AT content in the DUE region of *oriC*wt (25% GC). *oriC*2.0AT (10% GC) and *oriC*3.0AT (20% GC) were obtained through syn3 DNA amplification. Their amplification rates were accelerated compared with those of *oriC*wt. Although *oriC*wt could not amplify the AT-rich syn3 Region I (10 kb, 21% GC) in RCR, *oriC*2.0AT and *oriC*3.0AT were able to. This indicated that increasing the AT content in the DUE facilitated the amplification of an AT-rich mini-chromosome (Fig. 2B). AT-rich regions are prone to spontaneous unwinding [58], which may prevent the sufficient accumulation of topological tension required for *oriC* unwinding, and required *oriC* mutation more prone to unwind. *oriC*3.6AT was obtained through the random *oriC* library screening. It contained DUE mutations more AT-rich (15% GC) than the DUE of *oriC*3.0AT and showed a faster amplification rate than that of *oriC*3.0AT. Additionally, it was able to amplify another AT-rich syn3 region, Region II (10 kb, 23% GC), which *oriC*3.0AT could not amplify. Region II is also AT-rich and likely contains additional unknown sequences that inhibit RCR amplification, even when using *oriC*3.0AT. Notably, this region was lost during RCR amplification of the syn3 2AT region (50 kb, 24% GC), which supports the idea that Region II contains inhibitory sequences that interfere with RCR amplification. o*riC*5.6AT (10% GC) contains a designed DUE with a higher AT content than that of *oriC*3.6AT. Although its amplification rate was almost identical to that of *oriC*3.6AT, it facilitated DNA amplification at a lower temperature (16°C). Duplex DNA unwinding at *oriC* requires an elevated temperature [59], indicating that increasing the AT content in the DUE region enables unwinding, even under lower temperatures. The P1 nuclease assay also demonstrated the superiority of *oriC*5.6AT, which exhibited DNA unwinding at a lower DnaA concentration than *oriC*wt (Fig. 4D and E). The DUE-R region of *E. coli oriC*wt contains three repeated KAK motifs, increasing to five in *oriC*3.0AT and *oriC*3.6AT and to seven in *oriC*5.6AT (Figs 2A and 4A, gray bars). These continuous KAK motifs resemble the six consecutive NAN sequences of the DnaA-trio in the *B. subtilis oriC*, which function as ssDNA binding sites for DnaA oligomers assembled adjacent to DnaA boxes, thereby stabilizing the unwound region of DUE [44, 45, 58]. The increased number of KAK motifs may therefore stabilize the unwound DUE region through a mechanism analogous to the DnaA oligomer binding to the DnaA-trio sequences. This notion was further confirmed by the observation that extending the wild-type-derived KAK repeat to a total of five, without significantly altering the base composition, enhanced the RCR amplification rate (Fig. 4C; dueRKAK).

Mutations in the low-affinity DnaA boxes (τ2, I1, and I2) were selected together with DUE mutations in the random *oriC* library screening (Supplementary Fig. S2). These mutations rendered the DnaA box sequences closer to the DnaA consensus sequence. When these consensus-type mutations were introduced into *oriC*3.6AT, resulting in *oriC*4.0AT, the RCR amplification rate was slower (Fig. 3A and B). Perhaps the strengthened binding of DnaA to τ2, I1, and I2 may block the expansion of DUE unwinding. The DnaA box consensus-type mutations within τ2–I1–I2 (*oriC*3.4AT and *oriC*5.4AT) conferred a competitive advantage over *oriC*wt, particularly under DnaA-limiting conditions (Fig. 5C). These mutations would make *oriC* absorb more DnaA molecules, leading to the block of RCR amplification of the coexistent *oriC*wt because of the shortage of its available DnaA molecules (Fig. 5D). *oriC*3.4AT and *oriC*5.4AT also showed slightly lower amplification yield than that of *oriC*wt (Fig. 5C). This observation is likely due to the shortage of available DnaA molecules in these consensus-type *oriC* mutants, particularly at the late stage of RCR.

In our experiments, we obtained two types of mutants using different strategies: DUE mutants with a faster amplification rate and DnaA box consensus-type mutants with a competitive advantage against *oriC*wt. When the number of *oriC* templates is low and DnaA is abundant, the DUE mutants tend to be preferentially amplified. During RCR amplification, the number of *oriC* templates increases and thus the ratio of DnaA to *oriC* decreases. Under these conditions, the DnaA box mutants are more likely to be preferentially amplified over coexistent *oriC*wt. This observation is comparable to the *r*/*K* selection theory in ecology [46, 47]. The theory explains how different species allocate resources for reproduction and survival, with *r*-strategy species prioritizing rapid growth and reproduction, producing many offspring with minimal resource requirements, whereas *K*-strategy species prioritize stable populations, producing fewer offspring with higher resource requirements [46, 47]. The DnaA proteins are comparable to the resources, whereas the DNA molecules with different *oriC* mutations are like the species. Our *in vitro* evolutionary experiment demonstrates that the DUE AT-rich mutations were selected as *r-*strategy species, which grow faster (Fig. 3B), whereas the DnaA box consensus-type mutations were selected as a *K*-strategy species, which absorb higher DnaA molecules (Figs 3B and 5C and D). The *K*-selected DnaA box mutants had a more competitive advantage against *oriC*wt under limited resource (DnaA) conditions (Fig. 5C and D). Thus, the DnaA box mutants are selected when the population of *oriC* species increases and competition for scarce DnaA resources becomes more prominent.

## Supplementary Material

gkaf772_Supplemental_Files

## Data Availability

The data underlying this article are available in the article and in its online supplementary material.
