# Supplementary material for: Adaptive evolution of oriC through in vitro propagation of a mini-chromosome in RCR
Source: Nucleic Acids Res. 2025 Aug 11;53(15):gkaf772. doi: 10.1093/nar/gkaf772 (PMC12342881; doi:10.1093/nar/gkaf772)
Supplement: gkaf772_Supplemental_Files [file gkaf772_supplemental_files.zip › Supplementary_Figures.pdf]

Supplementary Figures

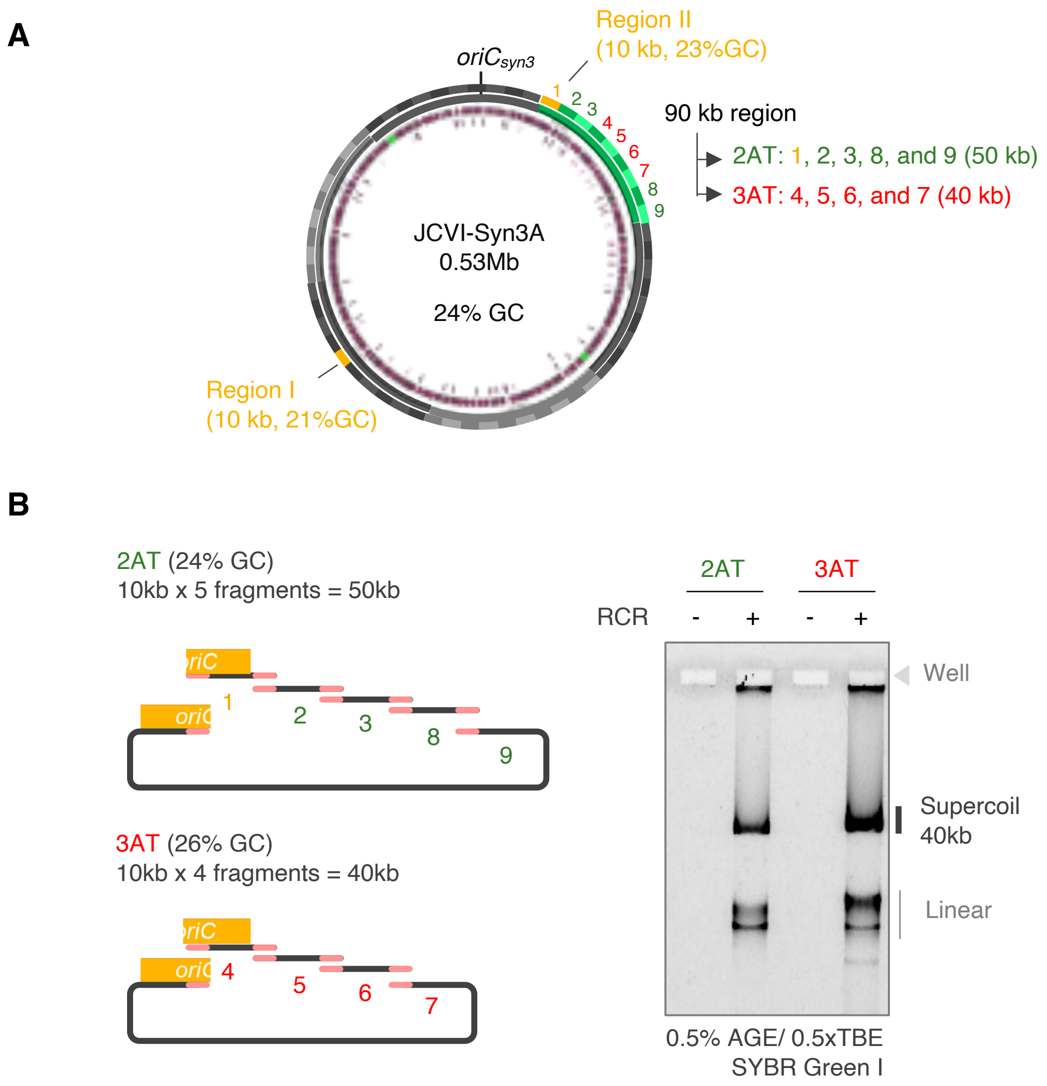

Figure S1. Construction of AT-rich mini-chromosome of JCVI-Syn3A using *E. coli oriC*wt. **A**. Schematic of JCVI-Syn3A (Syn3) genomic DNA. The position of 10 kb fragment regions used in this study, which were chemically synthesized by GenScript, are indicated by green and yellow dotted lines. **B**. RCR amplification using *E. coli oriC*wt of 2AT region (50 kb; fragments 1st, 2nd, 3rd, 8th, and 9th) and 3AT region (40 kb; fragments 4th, 5th, 6th, and 7th) from the 90 kb region shown in A. The analysis of the RCR amplified products is shown in the right panel.

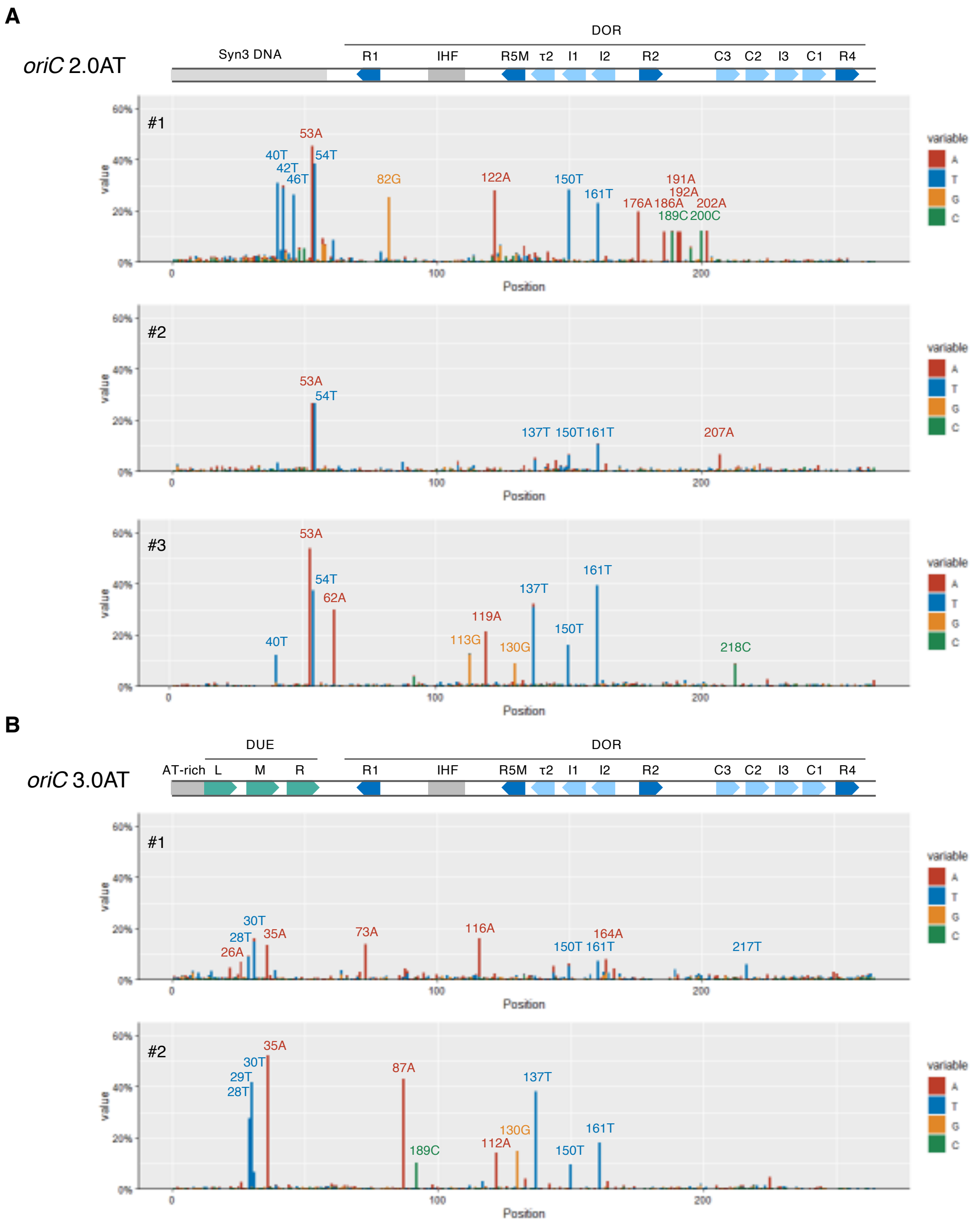

Figure S2. NGS analysis of RCR products amplified from ep-*oriC*2.0AT and ep-*oriC*3.0AT. NGS data were analyzed, and single-nucleotide polymorphisms (SNPs) were detected using Geneious software. SNPs data were graphed using R. **A**. Selected mutation from ep-*oriC*2.0AT. **B**. Selected mutation from ep-*oriC*3.0AT. These graphs represent the nucleotide position on the x-axis, starting from 70 base pairs upstream of the region just before the base of the R1 DnaA-box (as shown in Figure 2A), and the mutation rate per read count from NGS analysis on the y-axis.
