# Supplementary material for: Adaptive evolution of oriC through in vitro propagation of a mini-chromosome in RCR
Source: Nucleic Acids Res. 2025 Aug 11;53(15):gkaf772. doi: 10.1093/nar/gkaf772 (PMC12342881; doi:10.1093/nar/gkaf772)
Supplement: gkaf772_Supplemental_Files [file gkaf772_supplemental_files.zip › Supple_Data_manuscript.pdf]

Supplemental data

Materials and methods

DNA preparation

The primers used in this study are shown in Supplementary Table 1. All fragments were prepared using KOD One PCR master mix (TOYOBO, Osaka, Japan) and purified using Gel and PCR Clean-up kit (Macherey-Nagel, Düren, Germany). For real-time RCR analysis, P1 nuclease assay, amplification at low-temperature experiments, and competitive RCR experiments, 7 kb circular DNAs containing *oriC* were prepared as follows. The primers P1/P2 were used for PCR amplification of *oriC*wt, whose sequence is as follows:

5′-
gtcattttcacactataatgccgctatagtgagtcgtagtatgttgtacctaaagggatcctgggtattaaaaagaagatctatttatttag
agatctgttctattgtgatctcttattaggatcgcactgccctgtggataacaaggatccggcttttaagatcaacaacctggaaaggatc
attaactgtgaatgatcggtgatcctggaccgtataagctgggatcagaatgaggggttatacacagctgaggaactgaagagcagt
tgttctttggataactaccggttgatccaagcttcctgacagagttatccacagtagatcgcacgatctgtcagctcatttcctttaggtac
aacatactagaatatttgcctacagcctccttt -3′

The underlined section indicates the 245-bp minimal *oriC*. The resulting product was introduced into the pSV-β-Galactosidase vector (pSVβ) (6.8kb, 53%GC) (Promega, USA) and amplified using the Cell-Free Switching System (CFSS) (OriCiro, Tokyo, Japan), resulting in a mini-chromosome containing *oriC*wt (pSVβ-*oriC*wt). The mini-chromosomes containing *oriC*2.0AT, *oriC*3.0AT, and *oriC*3.4AT were constructed using the following primer pairs: P1/P4 and P2/P3, P1/P6 and P2/P5, and P1/P8 and P2/P7, respectively. These primers were then used for two separate PCR amplifications of the *oriC* region of the pSVβ-*oriC*wt DNA as a template. Subsequently, the two resulting fragments were assembled by overlapping PCR using primers P1/P2, and then introduced into the pSVβ vector using CFSS. The mini-chromosome containing *oriC*3.6AT was constructed using the primer pairs of P1/P10 and P2/P9, with *oriC*3.0AT DNA as a template. These two resulting fragments were assembled by overlapping PCR using primers P1/P2, and then introduced into the pSVβ vector using CFSS. *oriC*4.0AT was constructed as follows: Primer pairs P1/P8 and P2/P7 were used to amplify *oriC*3.6AT as a template, and the resulting fragments were assembled by overlapping PCR using P1/P2, which was then introduced into pSVβ using CFSS. The *oriC*5.4AT and *oriC*5.6AT were constructed using the primer pairs P1/P12 and P2/P11, and P1/P14 and P2/P13, respectively. These primers were used for amplifying *oriC*3.4AT and *oriC*3.6AT as templates, and the assembled fragments generated by overlapping PCR using P1/P2 were introduced into pSVβ using CFSS, resulting in *oriC*5.4AT and *oriC*5.6AT, respectively. *oriC*6.0AT was constructed as follows: Primer pairs P1/P14, P12/P13, and P2/P11 were used to amplify *oriC*4.0AT as a template, and the resulting fragments were assembled by overlapping PCR using P1/P2. The assembled product was then introduced into pSVβ using CFSS. The constructed and amplified mini-chromosomes containing *oriC* mutants using CFSS, as described, were purified using spin columns (QIAprep Spin Miniprep Kit, Qiagen, Hilden, Germany). The sequence of *oriC* mutants was confirmed by Sanger sequencing.

We constructed the 7 kb mini-chromosome containing poly-A or -T sequences in the DUE-M or DUE-R region as follows. *oriC*dueM9T, *oriC*dueM9A, *oriC*dueR13T, and *oriC*dueR13A were designed in the primer pairs P15/P16, P17/P18, P19/P20, and P21/P22, respectively. These primers were then used for inverse PCR amplification of the pSVβ-*oriC*wt DNA as a template. We also constructed *oriC*dueMAT, *oriC*dueR4AT, and *oriC*dueRKAK using primers P23/P24, P25/P26, and P27/P28, respectively, with the same template for inverse PCR amplification. Then, 100 µL of competent *E. coli* DH5α cells (DH5α high Champion, SMOBIO, Taiwan) were transformed with 1 µL of the resulting fragments directly, and the transformants were selected on Luria Bertani (LB) plate containing 50 µg/ml carbenicillin at 30°C. mini-chromosomes DNA containing *oriC*dueM9T, *oriC*dueM9A, *oriC*dueR13T, *oriC*dueR13A, *oriC*dueMAT, *oriC*dueR4AT, and *oriC*dueRKAK were purified from these transformant cells cultured at 30°C for 16 hours using spin-columns (QIAprep Spin Miniprep Kit, Qiagen, Hilden, Germany). The sequences of the *oriC* mutants were confirmed by Sanger sequencing.

DNA fragments of 90 kb region of JCVI-Syn3A (Syn3) (Accession number: CP016816, 425,400-510,660, Figure S1A) were chemically synthesized as nine of 10-kb fragments with 60-bp overlaps to adjacent fragments by GenScript Biotech Corp. (Nanjing, China). For the construction of AT-rich mini-chromosome of Syn3A (Figure S1B), DNA fragments of 2AT were prepared by PCR amplification using the following primers: P29/P30, P31/P32, P33/P34, P35/P36, and P37/P38 for the 1st, 2nd, 3rd, 8th, and 9th fragments, respectively. The left (backwards from the position at 119) and right (onwards from the position at 60, as shown in Figure S1B) of the *oriC* region were designed in the 1st and 9th fragments, with P25 and P38 used for amplification of these respective regions. The overlapping regions were utilized in DNA assembly using the cell-free cloning system (OriCiro Genomics, Tokyo, Japan). A DNA set of 3AT was prepared using the following primers: P39/P40, P41/P42, P43/P44, and P45/P46 for the 4th, 5th, 6th, and 7th fragments, respectively. To amplify 3AT in RCR, primers P47/P48 were used to amplify an *oriC* fragment containing sequences homologous to P39 and P46 of the 4th and 7th fragments, respectively.

As shown in Figures 2B and 3C, three 10 kb DNA fragments were prepared as follows: the lambda phage DNA region with 56% GC content (NIPPON GENE, Japan) was amplified using primers P49/P50; the Region I, with 21% GC content (Accession number: CP016816, positions 196,476–206,195; Figure S1A), was synthesized by GenScript Biotech Corp (Nanjing, China) and amplified using primers P51/P52; and the Region II, with 23% GC content, corresponding to the 1st fragment of the 90 kb region (Figure S1A), was amplified using primers P53/P54. *oriC* fragments were prepared by amplifying the *oriC* region using primers P55/P56 using mini-chromosomes containing *oriC* mutants described above as PCR template.
