# Supplementary material for: Adaptive evolution of oriC through in vitro propagation of a mini-chromosome in RCR
Source: Nucleic Acids Res. 2025 Aug 11;53(15):gkaf772. doi: 10.1093/nar/gkaf772 (PMC12342881; doi:10.1093/nar/gkaf772)
Supplement: gkaf772_Supplemental_Files [file gkaf772_supplemental_files.zip › Supple_Table.pdf]

Supplementary Table 1. Primers used in this study
Primers used in this study were listed.

Name	Sequence 5' → 3'
P1	GATCTAGGTGAAGATCCTTTTTGATAATCTCATGACCAAAATCCCTTAACGTGAGTTTTCGTCAT<br>TTTCACACTATAATG
P2	TTTTTGTTTGCAAGCAGCAGATTACGCGCAGAAAAAAAGGATCTCAAGAAGATCCTTTGAAAAG<br>GAGGCTGTAGGCAAATATTCT
P3	GGTAAATATATTTATATGATTATATTTATTATATTAACTATATTTATTGCTATTATCGCACTGCCCT<br>GTGGATAACAAGG
P4	TTAATATAATAAATATAATCATATAAATATATTTACCAATTCCATCATTTTGAAACTTTAGGTACAA<br>CATACTACGACTC
P5	TTAGAGATATGTTCTATTGTGATTATTATTAGGATCGCACTGC
P6	CCTAATAATAATCACAATAGAACATATCTCTAAATAAATAGATC
P7	GTGAATGATCTGTGATCCTGGACTGTATAAGCTGTGATCAGAATG
P8	ACAGCTTATACAGTCCAGGATCACAGATCATTCACAGTTAAT
P9	ATTTATATATATATTCTATTGTGATTATTATTAGGATCGCACTGC
P10	CCTAATAATAATCACAATAGAATATATATATAAATAAATAGATC
P11	AGGATCATTAACTGTGAATAATCTGTGATCCTGGACTGTA
P12	TACAGTCCAGGATCACAGATTATTCACAGTTAATGATCCT
P13	TTTATATATATATTATATTATTATTATTATTAGGATCGCACTGC
P14	CCTAATAATAATAATAATATAATATATATATAAATAAATAGATC
P15	TTTATTTAGTTTTTTTTTCTATTGTGATCTCTTATTAGGATCGCACTGCC
P16	ACAATAGAAAAAAAAACTAAATAAATAGATCTTCTTTTTAATACCCAGGA
P17	TTTATTTAGAAAAAAAAACTATTGTGATCTCTTATTAGGATCGCACTGCC
P18	ACAATAGTTTTTTTTTCTAAATAAATAGATCTTCTTTTTAATACCCAGGA
P19	TATTGTTTTTTTTTTTTTTGATCGCACTGCCCTGTGGATAACAAGGATCC
P20	GCGATCAAAAAAAAAAAAAACAATAGAACAGATCTCTAAATAAATAGATC
P21	TATTGTAAAAAAAAAAAAAAGATCGCACTGCCCTGTGGATAACAAGGATCC
P22	GCGATCTTTTTTTTTTTTTTACAATAGAACAGATCTCTAAATAAATAGATC
P23	TTTAGATATATTATTTATTGTGATCTCTTATTAGGATCGCACTGCCCTG
P24	GATCACAATAAATAATATATCTAAATAAATAGATCTTCTTTTTAATACC
P25	TATTGTGTATATATATTAGGATCGCACTGCCCTGTGGATAACAAGGATCC
P26	GCGATCCTAATATATATACACAATAGAACAGATCTCTAAATAAATAGATC
P27	TATTGTGTATTATTATTAGGATCGCACTGCCCTGTGGATAACAAGGATCC
P28	GCGATCCTAATAATAATACACAATAGAACAGATCTCTAAATAAATAGATC
P29	GATCCTTTCCAGGTTGTTGATCTTAAAAGCCGGATCC
P30	AATTAATTGGTGGAATTAAGTTTAGAGAAAGAAAAGTAATTAAAGATGCTATGGC
P31	AAAATGCCATAGCATCTTTAATTACTTTTCTTTC
P32	TGAGTACTTATAATTTTTGTGTTCAAGTGGTGGTCGTGGACGGAATTGAACC
P33	GTGTCGGCGGTTCAATTCCGTCCACGACCACCAC
P34	TTTATTATTAGGTTGATTTGTAATTATGTTTATTATTCCAAATGTTTGAAAATTATTTGTCCCGGGA<br>TGTTAGAAAATCCTAATATTAAAAAAACATATATTAGTGCTAG
P35	ACAAATAATTTTCAAACATTTGGAATAATAAACATAATTAC
P36	TTGGAGTTAACTTATTAAGAGAAGGATTAGATCTACCTG
P37	AAATACATACTAAACTAACTTCAGGTAGATC
P38	TCGCACTGCCCTGTGGATAACAAGGATCC
P39	AGTTAAAAAGAATTTTCTAGCACTAATATATG
P40	CTTTATTACTAGTTATAATAGTGCAATAGCTCAAAATAATAGTTTATTAGCAGCTCAATCACTAGC<br>TC
P41	GATTGAGCTGCTAATAAACTATTATTTTGAGCTATTGC
P42	TGTTCCTTTTAAGAATAGGTATATATGTTATTCCACTTTTTATTGCTTTGTTATTAAGTG
P43	CACTTAATAACAAAGCAATAAAAAGTGGAATAACATATATACC
P44	AGCTGGAAGATTTAAACTAAAACAAAAATTATC
P45	ACTCTATTTAAAATTCTATTTTTAATTGATAATTTTTGTTTTAGTTTAAATCTTCCAGCTTTAG
P46	TTTATTATTAGGTTGATTTGTAATTATGTTTATTATTCCAAATGTTTGAAAATTATTTGTTAAACC
P47	ACAAATAATTTTCAAACATTTGGAATAATAAACATAATTACAAATCAACCTAATAATAAACCCGGG<br>ATCTACTGTGGATAACTCTGTCAGGAAGCTTG
P48	ATGTTAGAAAATCCTAATATTAAAAAAACATATATTAGTGCTAGAAAATTCTTTTTAACTCCCGGG<br>TATTAAAAAGAAGATCTATTTATTTAGAGATCTG

P49	CTTTAGGTACAACATACTACGACTCACTATAGCGGCATTATAGTGTGAAAATGACCCACGCACG TTGTGATATGTAGATGATAATC
P50	ATCTGTCAGCTCATTTCCTTTAGGTACAACATACTAGAATATTTGCCTACAGCCTCCTTTGACGG TAATTTCTGCAACCGCAGCAAACTC
P51	CTTTAGGTACAACATACTACGACTCACTATAGCGGCATTATAGTGTGAAAATGACGCCGGATTT GTTATAAGTTTTGCAAC
P52	ATCTGTCAGCTCATTTCCTTTAGGTACAACATACTAGAATATTTGCCTACAGCCTCCTTTGATGAT TCAAAACTCTGACTAGTTGTTTTTTTATG
P53	CTTTAGGTACAACATACTACGACTCACTATAGCGGCATTATAGTGTGAAAATGACCATACTAGAT GTGTATAAGAGACAG
P54	ATCTGTCAGCTCATTTCCTTTAGGTACAACATACTAGAATATTTGCCTACAGCCTCCTTTAATTAA TTGGTGGAATTAAGTTTAGAGAAAG
P55	AAAGGAGGCTGTAGGCAAATATTCT
P56	GTCATTTTCACACTATAATG
